# Evaluation of the acute toxicity oral of carnauba powder (PCO-C) in mice C57BL/6

**DOI:** 10.1186/1758-5996-7-S1-A255

**Published:** 2015-11-11

**Authors:** Paula Alves Salmito Rodrigues, José Ytalo Gomes da Silva, Marcelo Oliveira Holanda, Carla Laine Silva Lima, Raquel Teixeira Terceiro Paim, Sandra Machado Lira, Natalia do Vale Canabrava, Mariana de Freitas Moreira, Juliana Barbosa Dantas, Thais Vital de Freitas, Julianne do Nascimento Sales, Bruno Bezerra da Silva, Erlândia Alves Magalhães Queiroz, Chayane Gomes Marques, Lia Magalhães de Almeida, Icaro Gusmão Pinto Vieira, Francisca Noélia Pereira Mendes, Rafaela Valesca Rocha Bezerra Sousa, Arnaldo Solheiro Bezerra, Maria Izabel Florindo Guedes

**Affiliations:** 1 Universidade Estadual do Ceará, Fortaleza, Brazil

## Background

Some plants associated with the treatment of diabetes are considered toxic because the hypoglycemic effect is often result of hepatotoxicity and β-adrenergic blockade. The Carnauba Powder produces a yellowish solid named PCO-C, which is predominantly composed of esters of cinnamic acid and has a chemical structure very similar to other compounds that have been described in the literature with a significant hypoglycemic effect, such as gamma-oryzanol and policosanol. Therefore, it is necessary to carry out acute toxicity tests to assess the PCO-C is safe for therapeutic use.

## Objective

The aim of the present work it was assess the acute toxicity of PCO-C in healthy mice.

## Materials and methods

To obtain the PCO-C, the same were extracted and isolated from the dust of unopened leaves of the carnaubeira, yielding a yellowish solid. Twelve C57BL/6, male mice between seven and eight weeks old, which they were kept under temperature 22 °C in light-dark cycle (12 in 12 h) and received standard chow and water ad libitum. The Ethics Committee on Animal Research approved the experimental protocol (no. 90/10) of this study. The mice were divided into 2 groups (n=6), saline and PCO-C, and was fasted for 4 h. After this period, they were administered, by gavage, saline (1 mL/Kg) and PCO-C solution in the dose of 2000mg/Kg. The animals had their behavior observed in the times 30, 60,90, 120, 150, 180, 210, 240, 270 and 300 min after the end of gavage. After the behavioral observation of the animals, their weight was daily measured for 12 days. After this period, the animals were euthanized to removal and analysis of the concerning weight vital organs to check for acute toxicity. The analysis of the significance differences between the data was performed using nonparametric test of the Mann Whitney, considering significant Results that had p <0.05.

## Results

**Figure 1:**
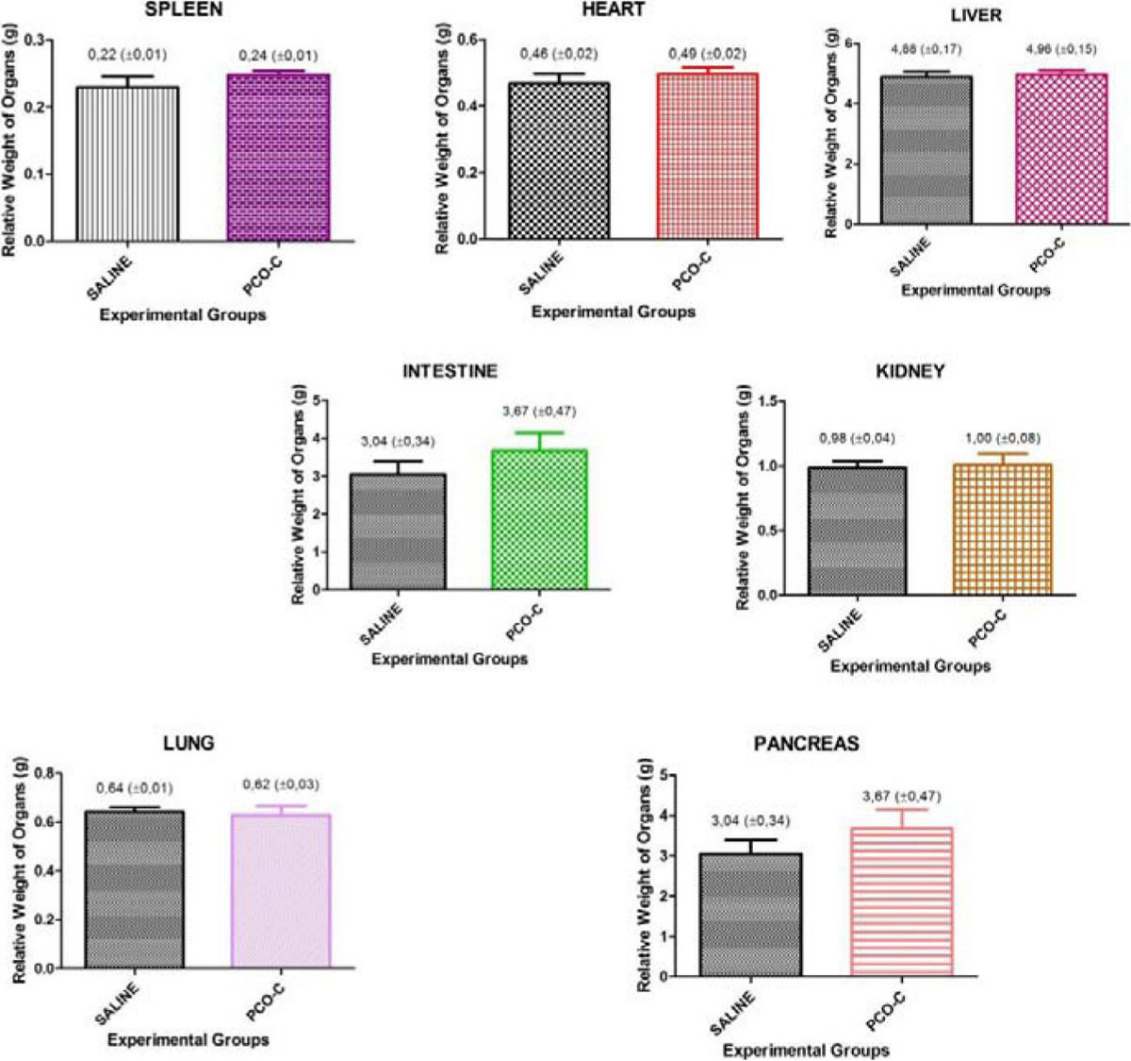
Result of the relative weight of organs.

No death was recorded and no significant change from the Hippocratic screening in the toxicity acute evaluation of the PCO-C in the dose of 2000 mg/Kg. Both groups had similar behaviors throughout the experiment. Also, there was no statistically significant difference in weight analysis concerning the organs (Figure 1), demonstrating the low toxicity of the PCO-C.

## Conclusion

The results indicate that the PCO-C does not have toxic effects and is safe for therapeutic use.

